# Supplementary material for: Prevalence and associated factors of skin disease among school-age children of rural district of eastern zone of Tigray, Ethiopia, 2024
Source: PLoS One. 2026 Feb 19;21(2):e0342768. doi: 10.1371/journal.pone.0342768 (PMC12919800; doi:10.1371/journal.pone.0342768)
Supplement: Attachment — Submitted filename: Response to Reviewers.docx [file pone.0342768.s002.docx]

**Response to Reviewers**

**PONE-D-25-26829**

**Title: Prevalence and associated factors of skin disease among school-age children of rural district of eastern zone of Tigray, Ethiopia, 2024.**

Dear Reviewers

We hope this message finds you well,

We wanted to express our sincere gratitude for your valuable feedback on our manuscript titled [Prevalence and associated factors of skin disease among school-age children of rural district of eastern zone of Tigray, Ethiopia, 2024]. Your thorough review and insightful comments have greatly contributed to improving the quality of the paper.

We have carefully considered all of your suggestions and made the necessary revisions accordingly. Two updated versions of the manuscript are attached for your review: one is highlighted in red to indicate and track the changes made, and the other is the revised and updated manuscript without highlights. Please find the point-by-point responses below, along with the revised and updated manuscript attached for your review.

Thank you once again for your time and effort. We look forward to any further feedback you may have.

Best regards,

Mulugeta Abreha

Corresponding Author

**Point by point response to Reviewers Comments: -**

| **Sr. No.** | **Reviewers' Comments** | **Response to Comments** |
| --- | --- | --- |
|  | **Academic Editor comments** |  |
| 1 | Please ensure that your manuscript meets PLOS ONE's style requirements, including those for file naming. The PLOS ONE style templates can be found at <https://journals.plos.org/plosone/s/file?id=wjVg/PLOSOne_formatting_sample_main_body.pdf> and <https://journals.plos.org/plosone/s/file?id=ba62/PLOSOne_formatting_sample_title_authors_affiliations.pdf> | We accept the comment and we made corrections according the PLOS ONE guideline. |
| 2 | We note that your Data Availability Statement is currently as follows: All relevant data are within the manuscript and in Supporting Information files. | The submission contains all raw data |
| 2 a) | If there are ethical or legal restrictions on sharing a de-identified data set, please explain them in detail (e.g., data contain potentially sensitive information, data are owned by a third-party organization, etc.) and who has imposed them (e.g., an ethics committee). Please also provide contact information for a data access committee, ethics committee, or other institutional body to which data requests may be sent. If data are owned by a third party, please indicate how others may request data access. | We accept the comment, and there are no ethical or legal restrictions on data access. We have already sent the data as supporting information |
| 3 | PLOS requires an ORCID iD for the corresponding author in Editorial Manager on papers submitted after December 6th, 2016. Please ensure that you have an ORCID iD and that it is validated in Editorial Manager. To do this, go to ‘Update my Information’ (in the upper left-hand corner of the main menu), and click on the Fetch/Validate link next to the ORCID field. This will take you to the ORCID site and allow you to create a new iD or authenticate a pre-existing iD in Editorial Manager. | I accept the comment, and I have registered in ORCID and obtained a new ID |
| 4. | Please include your full ethics statement in the ‘Methods’ section of your manuscript file. In your statement, please include the full name of the IRB or ethics committee who approved or waived your study, as well as whether or not you obtained informed written or verbal consent. If consent was waived for your study, please include this information in your statement as well. | We accept the comment, and we include the full name of IRB in the Methods section. |
| 5. | Please include a copy of Table 5 which you refer to in your text on page 22. | The comment is well taken and we make revision accordingly |
| 6 | If the reviewer comments include a recommendation to cite specific previously published works, please review and evaluate these publications to determine whether they are relevant and should be cited. There is no requirement to cite these works unless the editor has indicated otherwise. | We accept the comment, and We make revision |
|  | **Reviewer#1** |  |
|  | **General comments:** |  |
| 1 | This study provides an overview of skin conditions among school-aged children but does not specify particular skin diseases. The findings mainly describe general skin conditions such as erosion, which may potentially result from trauma rather than a defined dermatological disorders. They also mention handwashing as associated with skin diseases, but the specific types of skin conditions are not specified. Furthermore, they did not provide detailed information on nail or hair diseases, which are typically classified as conditions. Therefore, the data on the prevalence of skin conditions is somewhat limited and lacks detail. Although trained medical staffs, such as nurses, can perform initial physical examinations, confirmation by licensed physicians is recommended. It would also be beneficial to categorize skin diseases into specific groups, such as infections (e.g., scabies), trauma-related issues, and dermatoses like atopic dermatitis or seborrheic dermatitis, to improve clarity and diagnosis accuracy. | We accept the comment and we make revision accordingly |
| 2 | Additionally, the associated factors for skin conditions is limited, with most focusing on caregivers' conditions. They should also inquire about participants' birth history, vaccination status, or activities involving water or sun exposure that may be related to skin diseases.  For presenting numbers, the authors should ensure consistency by using two decimal places (e.g., 5.00) for clarity and uniformity.  They should include abbreviations under the table and also define them in the abstract when they first appear. | We accept the comment, and We make modification accordingly. |
|  | **Reviewer #2:** |  |
| 1 | 1. professional English editing  - Improving tables 2 & 3 as follows:  2. under the column Variables: instead of "Family History of skin disease" change to: "Positive family history of skin disease", then remove the column Response, and keep the Frequency and Percentage for the answer "Yes" only. 3. please do the same for all other variables. 4. for variable with responses other than yes/no, move the responses and list them in the same column as the variables | We accept the comment, and we have made changes based on the comments |
| 2 | Adding the diagnoses rather than the morphology of skin diseases under the title "The common types of skin diseases identified". I think the morphology alone may not be accurate. Adding the diagnosis will help making any association between independent and dependent variables more reasonable. E.g. kids washing their hands frequently are expected to have atopic dermatitis. Kids who don’t wash their hands are expected to have infections or infestations. Kids with a positive family history that is recent are expected to have infection or infestation. | The comment is well taken and we make revision |
| 3 | - Correcting the number of the table titled "Bivariate analysis of factors associated with skin disease among school-age children of Rural District of Eastern Zone, Northern Ethiopia, 2024" from 3 to 4 - Correcting the number of the table titled "Multivariate analysis of factors associated with skin disease among school-age children of rural district of eastern zone, northern Ethiopia, 2024" from 4 to 5. - Removing figure 1, it is not necessary. | We accept the comment, and we make correction accordingly |

We would be glad to respond to any further questions and comments that you may have.
